# Precision Target Discovery for Migraine: An Integrated GWAS-eQTL-PheWAS Pipeline

**DOI:** 10.3390/molecules30193921

**Published:** 2025-09-29

**Authors:** Xianting Liu, Qingming Liu, Haoning Zhu, Xiao Zhou, Xinyao Li, Ming Hu, Fu Peng, Jianguang Ji, Shu Yang

**Affiliations:** 1 Key Laboratory of Drug-Targeting and Drug Delivery System of the Education Ministry and Sichuan Province, Sichuan Engineering Laboratory for Plant-Sourced Drug, Sichuan Research Center for Drug Precision Industrial Technology, West China School of Pharmacy, Sichuan University, Chengdu 610041, China; liuxt_1122@163.com (X.L.); liuqingming@scu.edu.cn (Q.L.); zhuhn_2019@163.com (H.Z.); scuzhouxiao@163.com (X.Z.); 2021151660222@stu.scu.edu.cn (X.L.); huming@scu.edu.cn (M.H.); 2 Faculty of Health Sciences, University of Macau, Macau SAR, China

**Keywords:** Mendelian randomization, migraine, druggable genes, molecular science

## Abstract

Migraine is a complex neurological disorder that severely compromises quality of life. Current therapies remain inadequate, creating an urgent need for precision medicine approaches. To bridge this gap, we integrated genome-wide association studies (GWASs) and multi-tissue expression quantitative trait loci (eQTL) data. Using Mendelian randomization (SMR/HEIDI) to identify putatively causal genes, followed by colocalization analysis, protein–protein interaction networks, and gene enrichment, we prioritized druggable targets. Phenome-wide association studies (PheWASs) further assessed their potential safety profiles. We identified 31 migraine-associated genes in whole blood, 20 in brain tissue, and 9 genes shared by both whole blood and brain regions. Among 13 druggable genes identified from the DGIdb and supporting literature, 10 passed colocalization validation. Eight genes (TGFB3, CHRNB1, BACE2, THRA, NCOR2, NR1D1, CHD4, REV3L) showed interactions with known drug targets, enabling the computational prediction of 41 potential repurposable drugs. Based on target druggability, PPI (protein–protein interaction) and favorable PheWAS profiles, NR1D1, THRA, NCOR2, and CHD4 are prioritized for drug development. Additionally, MICU1, UFL1, LY6G5C, and PPP1CC emerged as novel pathophysiological factors. This study establishes a multi-omics framework for precision migraine therapy, translating genetic insights into clinically actionable targets.

## 1. Introduction

Migraine is a common neurological disorder affecting approximately 14% of the global population [1], characterized by recurrent moderate-to-severe headaches, often accompanied by nausea, vomiting, photophobia, and phonophobia [2]. This disease not only significantly affects patients’ quality of life, but also leads to a substantial socioeconomic burden [3]. Although medications such as triptans and calcitonin gene-related peptide (CGRP) inhibitors are available [4,5], many patients experience suboptimal efficacy or intolerable side effects [6]. This highlights the urgent need to discover novel therapeutic targets and drugs to improve the efficacy of migraine treatment and reduce side effects [7].

Traditional drug target discovery relies on labor-intensive and time-consuming processes, including preclinical validation and clinical translation, which may span years and significantly delay therapeutic innovation [8,9]. Omics technologies, particularly genomics, have revolutionized this landscape by enabling the unbiased identification of disease-associated genetic variants and molecular signatures. Genome-wide association studies (GWASs), for instance, have identified more than 40 loci associated with migraine, including genes involved in ion transport and neuronal excitability, such as CACNA1A [10,11,12]. This gene-centric approach not only accelerates the discovery of candidate targets, but also enhances precision by reducing the reliance on empirical screening [8]. Mendelian randomization (MR) has emerged as a powerful tool for causal inference in observational studies, leveraging genetic variants as instrumental variables (IVs) to mitigate confounding and reverse causation [13]. By mimicking the random allocation of alleles during meiosis, MR provides quasi-experimental estimates of the causal relationships between exposures (e.g., gene expression) and outcomes (e.g., migraine) [14,15]. Summary-data-based MR (SMR) is one of MR analysis tools based on summary data, which can efficiently analyze large-scale summary data to test the pleiotropic association between gene expression levels and complex traits of interest [16,17]. SMR and heterogeneity in dependent instruments (HEIDI) analysis can effectively test whether the magnitude of the effect of single nucleotide polymorphisms (SNPs) on phenotypes is mediated by gene expression [16]. For instance, SMR has successfully elucidated associations between host OS gene expression and gut microbiota [18,19], between the composition of gut microbiota and sepsis [20], as well as between immune-related and inflammation-related genes and intracranial aneurysms and their subtypes [21], demonstrating its utility in identifying candidate therapeutic targets.

However, the validity of MR conclusions hinges critically on four core assumptions of the instrumental variable (IV) framework [22,23,24]: (1) Relevance—the IV must be strongly associated with the exposure [23]; (2) Independence—the IV must be independent of confounders; (3) Exclusion restriction—the IV must influence the outcome exclusively through the exposure [23,25]; and (4) Monotonicity—the direction of the IV’s effect must be consistent across individuals. The IV pleiotropy (single SNP affecting multiple traits) violates this assumption, necessitating sensitivity analysis, such as MR–Egger regression [24]. Moreover, single MR analysis is susceptible to biases arising from small sample sizes, population stratification, and horizontal pleiotropy [26]. To address these challenges, this study integrates multi-source expression quantitative trait locus (eQTL) datasets, derived from large-scale consortia, such as GTEx, eQTLGen, and PsychENCODE, with migraine GWAS summary data [27]. Complementing genomic approaches, we further incorporate protein–protein interaction (PPI) network analysis [28] and phenome-wide association studies (PheWASs) [29] to strengthen target discovery. PPI networks map functional protein connections, facilitating the identification of hub proteins whose dysregulation [28] may drive migraine pathogenesis, thereby narrowing the candidates to high-impact molecules. Meanwhile, PheWASs expand beyond disease-specific GWASs by linking genetic variants to a broad spectrum of phenotypes [29], aiding in the identification of pleiotropic genes that influence both migraine and its comorbidities. By integrating SMR with HEIDI testing, colocalization, and PheWAS analysis, we aim to (1) systematically prioritize migraine-associated genes through causal inference; (2) validate the findings across independent cohorts to ensure robustness; and (3) pinpoint druggable targets with high translational potential. Our integrated pipeline ultimately seeks to accelerate precision target discovery and inform future migraine therapeutics.

## 2. Results

### 2.1. SMR Main Analysis

SMR analysis results using genetic tool data, including target gene expression in whole blood from the **eQTLGen Consortium**, prefrontal cortex cis-eQTL data from the **PsychENCODE project**, and data from 13 brain regions (amygdala, anterior cingulate cortex, caudate nucleus, cerebellar hemisphere, cerebellum, cerebral cortex, frontal cortex, hippocampus, hypothalamus, nucleus accumbens, putamen, spinal cord (as part of the central nervous system), and substantia nigra) [30,31], and whole blood of the **GTEx V8 project**, are presented in Supplementary Tables S1–S3.

Using FDR < 0.05 and HEIDI > 0.05 as threshold criteria, 60 genes (after deduplication) were identified as significantly associated with migraine. Among these, 31 were expressed in whole blood, 20 in brain regions, and 9 were shared between whole blood and brain regions, as detailed in Table 1.

In order to validate the robustness and reproducibility of the primary MR results, we used two external datasets (BrainMeta v1 and BrainMeta v2) to replicate the analysis. The significant results of the main analysis, cross-validated by ≥2 databases, are presented in Supplementary Table S4a,b.

### 2.2. Identification and Profiles of Screened Druggable Genes

**Figure 1:**
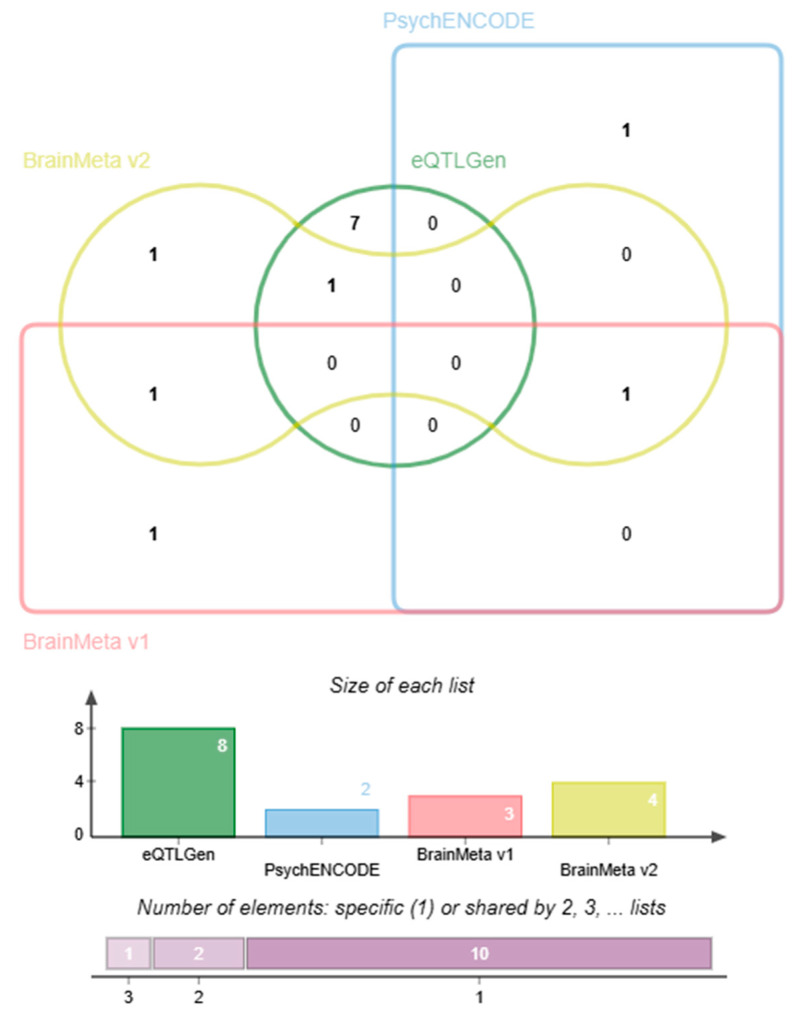
The summary results of druggable genes screened out from the significant result genes obtained from SMR analysis.

To clarify whether there are potential repurposable drugs, we extracted the data on druggable genes from the studies of Wei-Ming Su (2023) [32] and Kang-Fu Yin (2024) [33], and screened out eight blood genes (AURKC, CHD4, GBA2, HVCN1, NCOR2, NR1D1, TGFB3, THRA) and five brain region genes (BICD1, IPO8, BACE2, REV3L, CHRNB1) (as shown in Figure 1). The SMR results of the 13 druggable genes are presented in Table 2. Among them, three genes (AURKC, BACE2, IPO8) were validated through independent validation in at least two databases (Supplementary Table S4b).

### 2.3. MR Analysis Results Based on Multiple Methods

**Figure 2:**
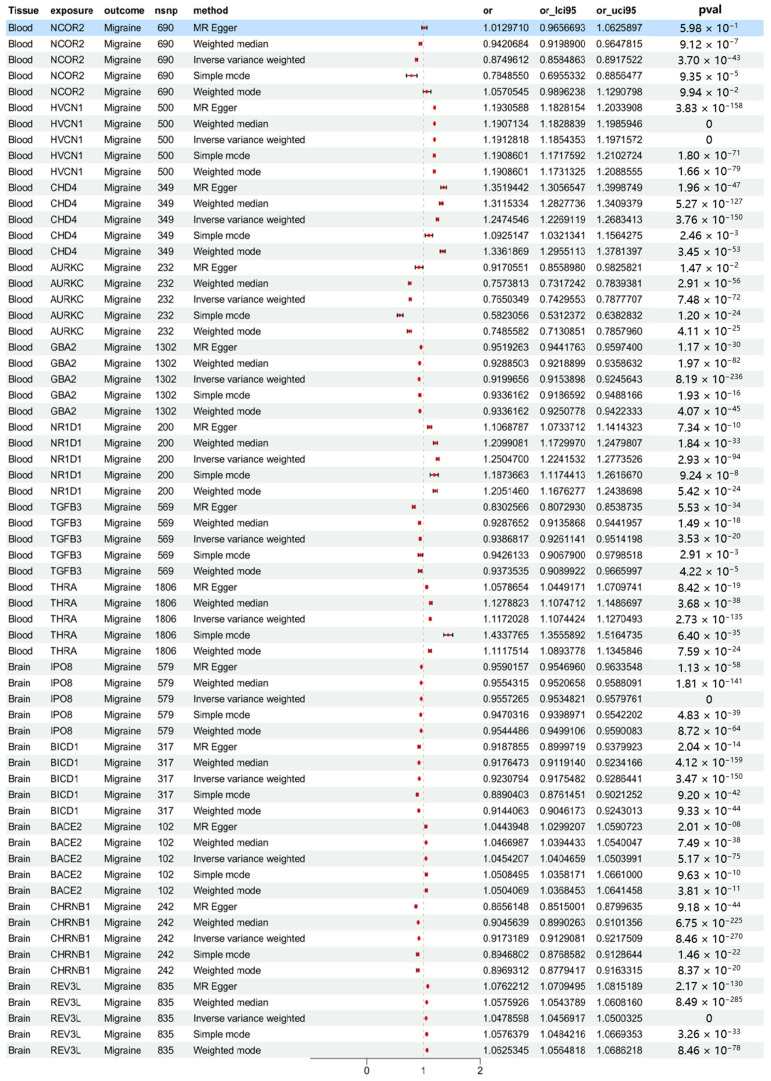
Forest plot of the estimated mendelian randomization effects and 95% confidence intervals for the causal associations of 13 druggable genes with migraine. Dots represent OR point estimates of the effect and lines represent 95% confidence intervals. FDR, the Benjamini–Hochberg method was used to control the error detection rate; FDR < 0.05 indicates the result is significant.

**Figure 3:**
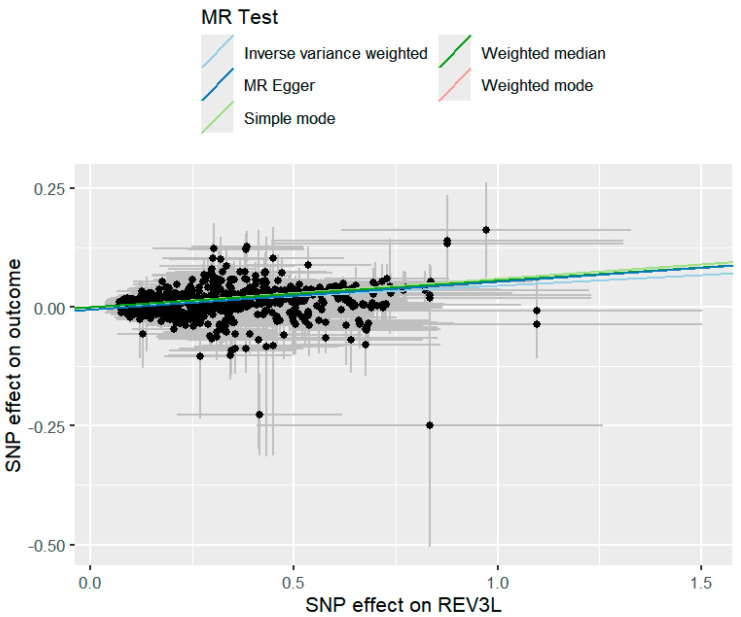
Scatter plot of the causal relationship between the instrumental variable (taking REV3L as an example) and the GWAS of migraine, with its slope corresponding to the estimated causal relationship. The intercept represents horizontal pleiotropy (the closer it is to 0, the smaller the horizontal pleiotropy). The scattered distribution indicates heterogeneity.

To assess the consistency of causal estimates, five MR methods were used to validate the significant genes identified by SMR analysis, including inverse variance weighted, weighted mode, simple mode, weighted median, and MR Egger (Supplementary Table S5). Data analysis was performed using the “TwoSampleMR”, “MR-PRESSO”, and “MendelianRandomization” packages. Data cleaning and statistical analyses were conducted using R version 4.2.2 (https://www.r-project.org/, accessed on 4 May 2025). As shown in Figure 2, the results demonstrate that 13 druggable genes that were significantly associated with migraine were simultaneously validated by multiple MR methods. For NCOR2, which did not pass the Egger intercept or global test, the “MR-PRESSO outlier test” and “MR-PRESSO distortion test” were used to verify the consistency of the results after removing outlier SNPs (as shown in Figure 3).

### 2.4. Colocalization Analysis of Druggable Genes

**Figure 4:**
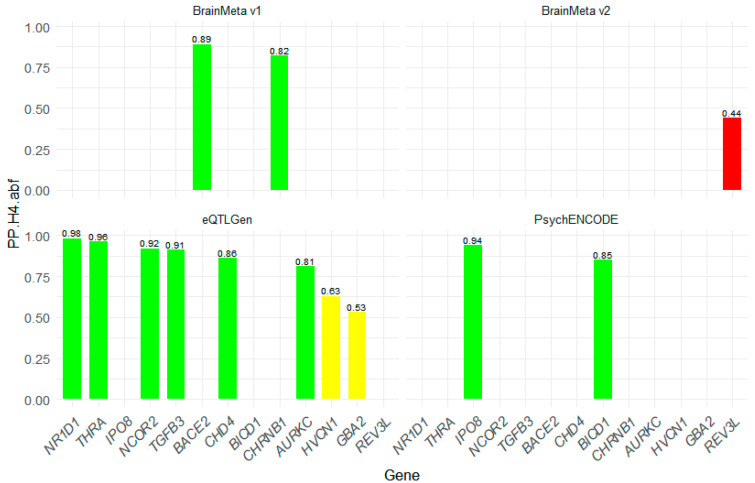
The bar chart of colocalization analysis results of druggable genes. The vertical axis represents the PP.H4.adf values (divided into three intervals). PP.H4.adf > 0.8 indicates that the GWAS signal and eQTL may originate from the same causal variation, suggesting that this gene may be a potential pathogenic gene. The higher the PP.H4.adf value, the better. PP.H4.adf < 0.5 suggests that the causal relationship between this gene and migraine needs further verification.

To further identify the pleiotropic association between druggable genes and migraine, we performed a colocalization analysis to confirm that the eQTL and GWAS signals likely originate from the same causal variant. Colocalization analysis was conducted on 13 significant druggable genes, and we found that 10 genes passed the colocalization analysis. The posterior probability results show that the causal variation probability shared by the three genes with migraine is relatively low (GBA2 (PP.H4.abf = 0.53), HVCN1 (PP.H4.abf = 0.63), REV3L (PP.H4.abf = 0.44)), which is insufficient to support the causal effect of this gene on migraine (as shown in Figure 4).

### 2.5. Exploration of the Druggability Potential of Genes

**Figure 5:**
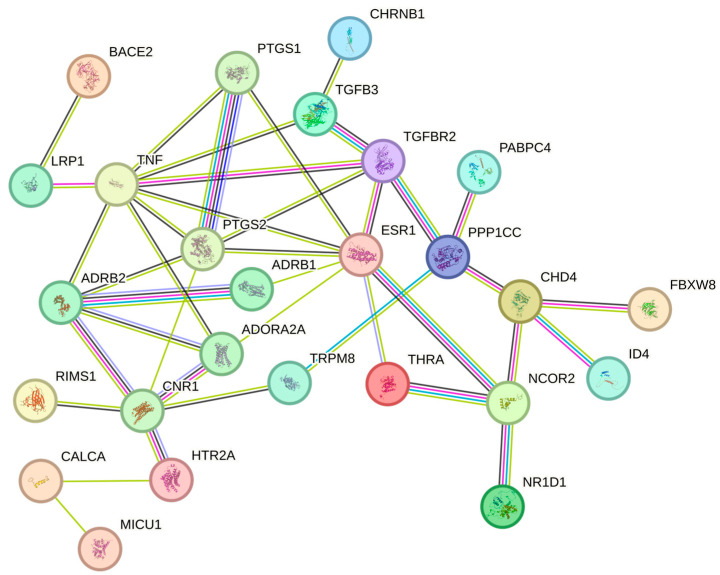
Protein–protein interaction network diagram (interaction score > 0.4). The density of the thin lines represents the degree of protein association (the denser the thin lines, the higher the degree of association).

**Figure 6:**
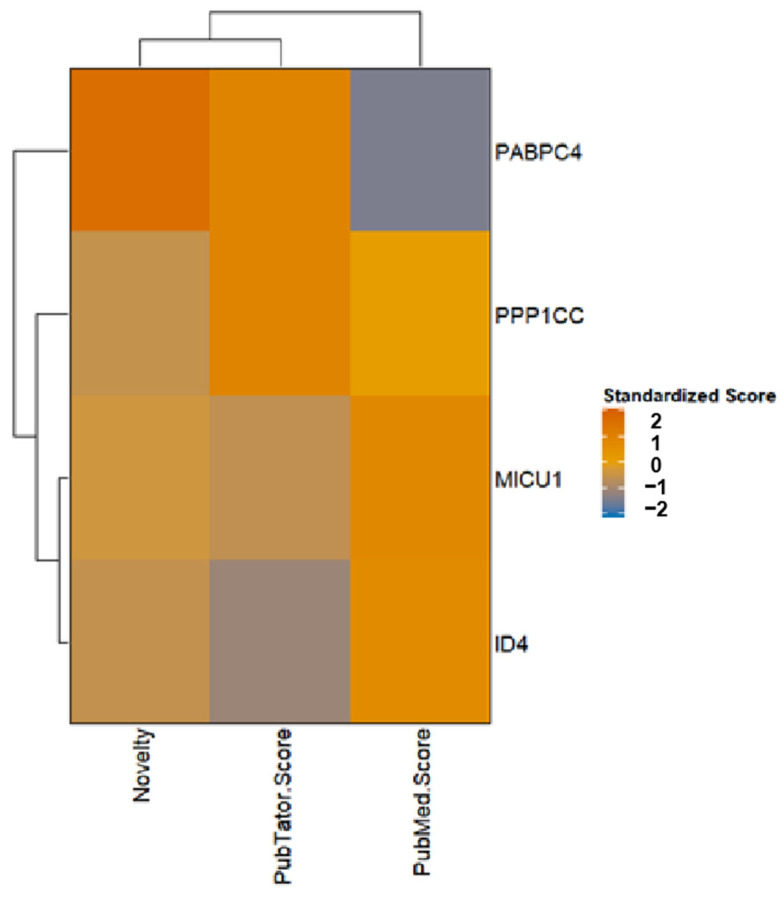
Heat map of key undruggable genes. The standardized scores for Novelty, PubTator Score, and PubMed Score are presented. The heat map represents the size of the value through the depth of color. The color bar provides a range reference for standardized scores (the darker the color (close to red), the higher the score; the lighter the color (close to blue), the lower the score).

We obtained 74 novel migraine-related targets from Pharos v3.19 (https://pharos.nih.gov/), 42 of which are already in clinical development (Supplementary Table S6). For instance, targets such as TRPM8, TNF, and ESR1 have been used in clinical drug development. To further explore the pathological mechanisms of migraine and the druggability of key target genes identified by multi-tissue SMR analysis, we used the STRING database for PPI network analysis. The organism was set to Homo sapiens, and the minimum interaction score was defined as medium confidence (0.40). The results show (as shown in Figure 5) that seven blood genes (THRA, NCOR2, NR1D1, CHD4, TGFB3, PABPC4, PPP1CC) and four brain genes (BACE2, CHRNB1, ID4, MICU1) are moderately associated with ten novel migraine targets (interaction score > 0.4), among which seven are druggable genes (THRA, NCOR2, NR1D1, CHD4, TGFB3, BACE2, CHRNB1). The results of evaluating the drug development potential of the remaining four genes (MICU1, ID4, PPP1CC, PABPC4) are shown in Figure 6, with PPP1CC having the highest standardized score. MICU1 had the highest PubMed score (354.26), followed by ID4. The target development level of both was “Tbio”, indicating no related drug development has been conducted yet (specific scores are provided in Supplementary Table S7).

### 2.6. Enrichment Analysis

**Figure 7:**
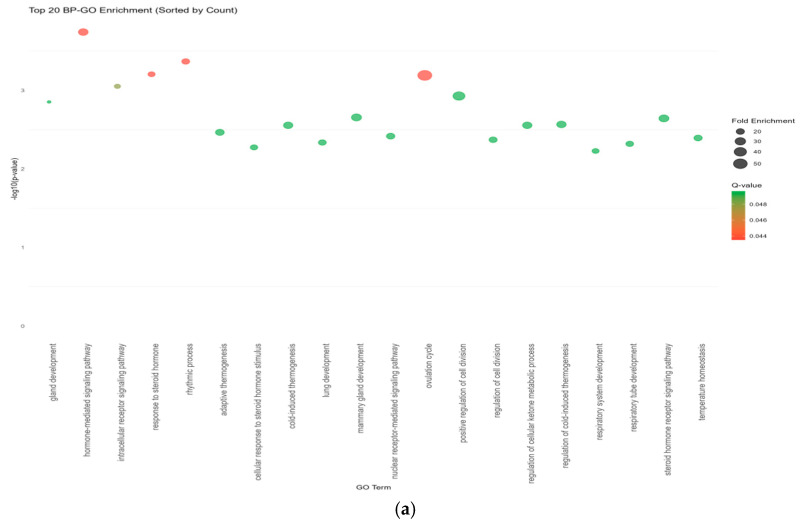

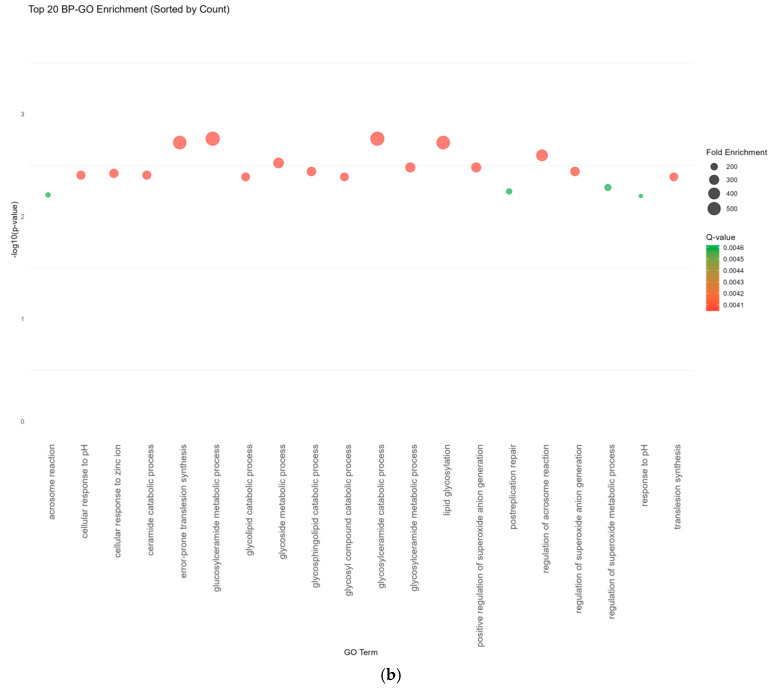
(**a**) GO (Gene Ontology) functional enrichment analysis diagram (druggable genes). (**b**) GO (Gene Ontology) functional enrichment analysis diagram (undruggable genes). It shows the enrichment degrees of the top 20 biological processes, and the horizontal axis represents different biological processes (GO term); the vertical axis represents −log10(*p*-value), and the larger the value, the higher the enrichment significance of this biological process. The size of the bubbles indicates the enrichment degree of the biological process (the larger the bubbles, the higher the enrichment degree of the biological process). The color of the bubbles represents the Q-value (corrected p-value) (the smaller the Q-value, the higher the significance of enrichment).

The results of the Gene Ontology enrichment (GO enrichment) analysis show that the 10 migraine-related druggable genes are primarily enriched in gland development, intracellular receptor signaling pathways, and responses to steroid hormones, as well as biological processes, such as steroid hormone biosynthesis (Figure 7a). These findings suggest that the pathogenesis of migraine may be closely linked to endocrine regulation, cellular signal transduction, and biological rhythms, providing critical insights for further elucidating the molecular mechanisms of migraine and developing novel therapeutic strategies.

The GO enrichment analysis of the drug potential genes (REV3L, GBA2, HVCN1) revealed that they were significantly enriched in multiple metabolic processes (e.g., glycosylceramide metabolism, glycolipid catabolic metabolism, lipid glycosylation) and cellular responses (e.g., cellular response to pH, acrosome reaction, and regulation of superoxide anion generation) (Figure 7b). Although the colocalization analysis of these genes with migraine GWASs did not reach statistical significance (PP.H4.abf < 0.8), their enrichment in specific biological processes suggests potential involvement in migraine pathogenesis through indirect mechanisms, thereby providing novel potential therapeutic targets for migraine treatment.

### 2.7. PheWAS Analysis

**Figure 8:**
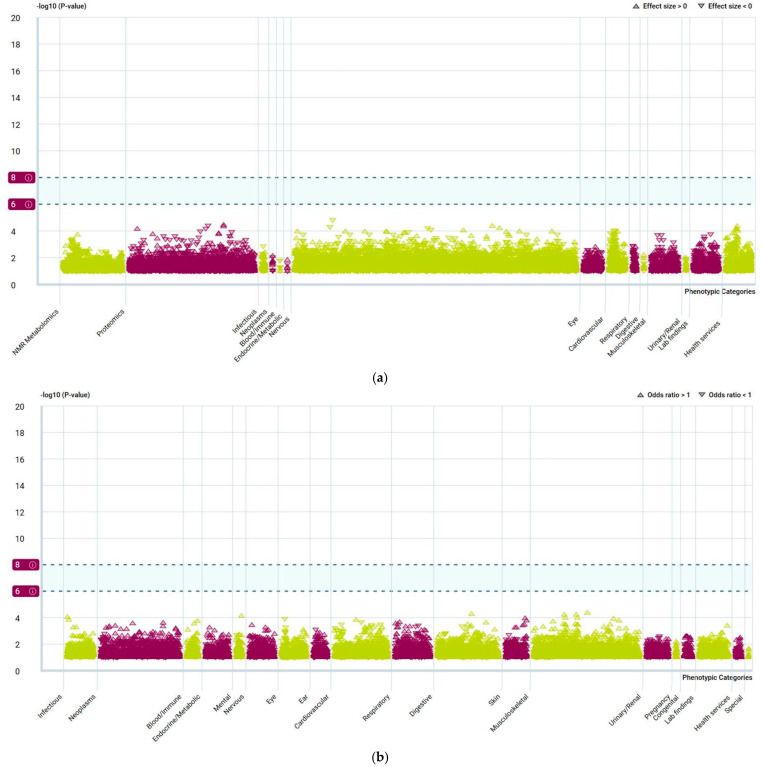
(**a**). Manhattan plot of NCOR2 (Continuous traits). (**b**). Manhattan plot of NCOR2 (binary traits). The vertical axis represents the −log_10_ (*p*-value); the horizontal axis represents phenotypic categories; the triangular direction represents the OR effect value (the upper triangle indicates the effect size > 0).

PheWAS analysis further confirmed the non-horizontal pleiotropy of these genes. As shown in Figure 8, when we take NCOR2 as an example, both continuous and binary traits fall within the threshold range (−log_10_(*p*-value) < 8).

### 2.8. Potential Targeted Drug Prediction

**Figure 9:**
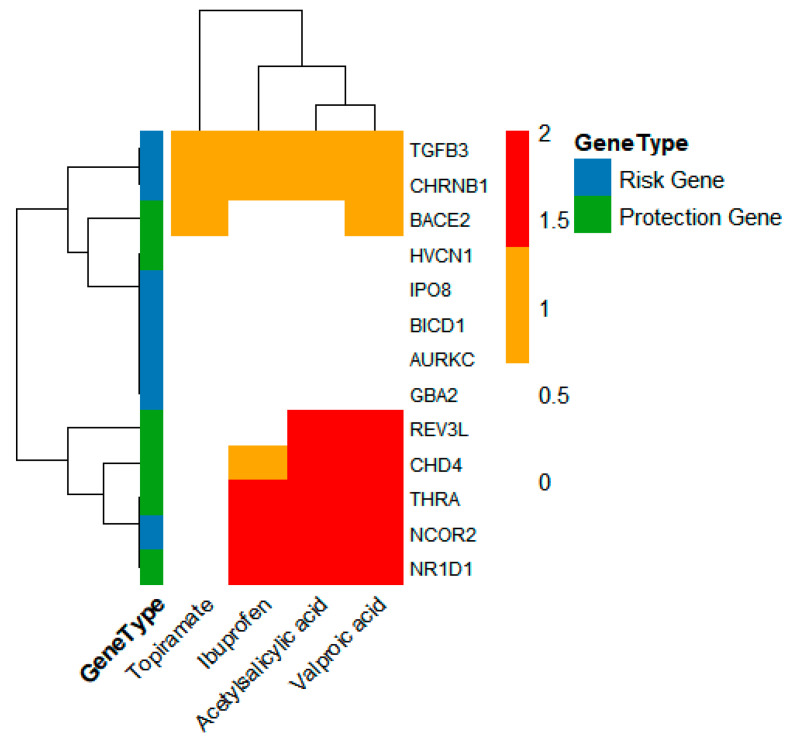
Protein–protein interactions between key druggable genes and first-line medications for migraine. This graph shows the PPI between 7 risk genes and 6 protective genes and four first-line medication targets for migraine. Red indicates high reliability and yellow indicates moderate reliability.

First-line drug target information for the prevention and acute treatment of migraine was obtained from the DrugBank database (v5.1.7) (encompassing 53 targets; see Supplementary Table S8), and the STRING database (v11.5) was used for network construction. Interaction confidence levels were classified as medium (0.40) or high (0.70). A gene interaction network was constructed between the 13 potential drug targets and 53 known migraine therapeutic targets. As shown in Figure 9, among the 13 identified potential drug targets, 8 (TGFB3, CHRNB1, BACE2, THRA, NCOR2, NR1D1, CHD4, REV3L) interacted with the targets of two acute migraine treatments (aspirin, ibuprofen) and two preventive drugs (topiramate, valproic acid). Among these, five (THRA, NCOR2, NR1D1, CHD4, REV3L) exhibited high-confidence associations with first-line migraine therapeutic targets.

**Figure 10:**
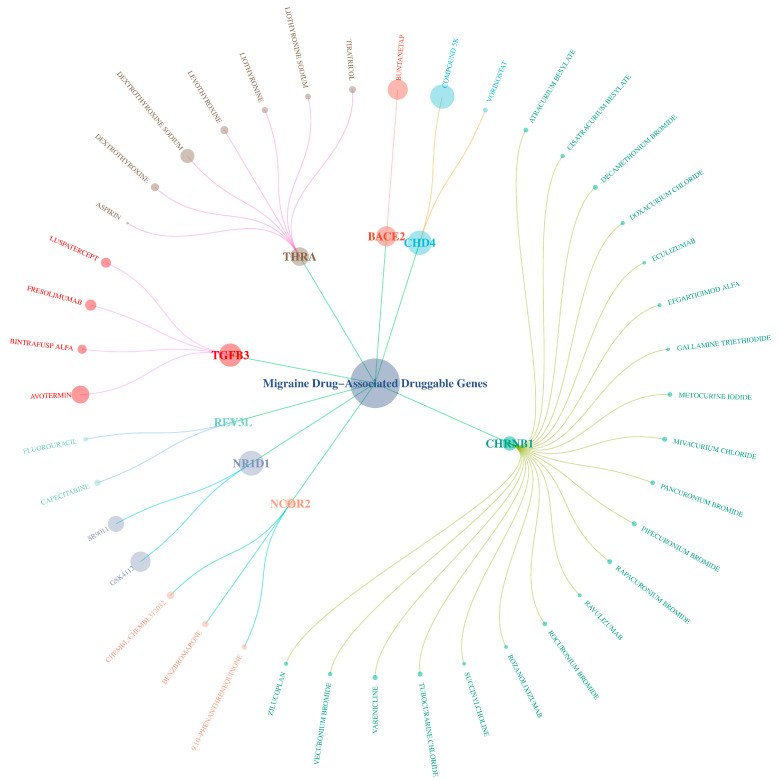
Graph of the association between genes and drugs. The nodes of different colors in the figure represent different drug-target genes related to migraine treatment (such as NCOR2, THRA, CHD4, TGFB3, etc.), and the labels on the nodes connected to the gene nodes (such as Dextrothyroxine Sodium, Levothyroxine) indicate the specific drug names. The size of the node indicates the degree of association between this gene and the drug (the larger the node, the higher the association).

Existing drugs (including marketed and unapproved drugs) related to the potential drug targets were retrieved via the DGIdb (https://dgidb.org) and interaction scores were calculated to evaluate the association strength between the drugs and targets (larger nodes in the network indicate higher interaction scores) (specific data are provided in Supplementary Table S9). The results show (as shown in Figure 10) that these 8 druggable genes are associated with 41 existing drugs, revealing that drugs such as 4 TGFB3 inhibitors and 7 THRA agonists may have off-label use for treating or preventing migraine. Furthermore, THRA exhibits a significant interaction (interaction score of 0.168) with aspirin and is the only gene associated with marketed drugs for migraine.

## 3. Discussion

In this study, we leveraged GWAS summary statistics from over 870,000 European individuals [12] and integrated multi-source, multi-tissue eQTL data using SMR. This enabled the identification of multiple migraine-associated genes with putative causal roles, laying a foundation for a mechanistic insight and target-based therapy development. This further provides an important basis for the pathological mechanism analysis and precise treatment of migraine.

### 3.1. Causal Associations and Biological Mechanisms

Through SMR analysis, we found that migraine-associated genes are primarily concentrated on Chromosome 6 (UFL1, LY6G5C, REV3L, etc.) and Chromosome 12 (PPP1CC, IPO8, BICD1, NCOR2, CHD4, etc.). Brain region localization analysis further revealed that the cerebellum and cerebellar hemispheres are key regions associated with migraine, enriched for genes such as UFL1 and LY6G5C. This finding aligns with previous neuroimaging studies reporting abnormalities in these brain regions among migraine patients [34], suggesting that these chromosomal regions may influence migraine pathology by regulating gene expression in specific neural tissues.

Notably, core genes such as CHD4 [35,36] and PPP1CC [37] play central roles in the PPI network and are implicated in critical biological processes, including epigenetic regulation, neural development, and synaptic plasticity. For instance, CHD4, as a core component of the chromatin remodeling complex, regulates epigenetic modifications that may influence neuronal differentiation and neuroinflammation [38,39], the latter being recognized as an important pathological hallmark of migraine [38]. Additionally, PPP1CC participates in neural precursor cell differentiation and synaptic transmission by modulating the phosphorylation status of cyclins [40]. Aberrant expression of PPP1CC may contribute to neuronal damage and synaptic remodeling in migraine patients [41]. Collectively, these detailed analyses of gene functions provide new insights into the neurobiological mechanisms underlying migraine.

### 3.2. Translational Implications: Drug Targets and Repurposing

**Figure 11:**
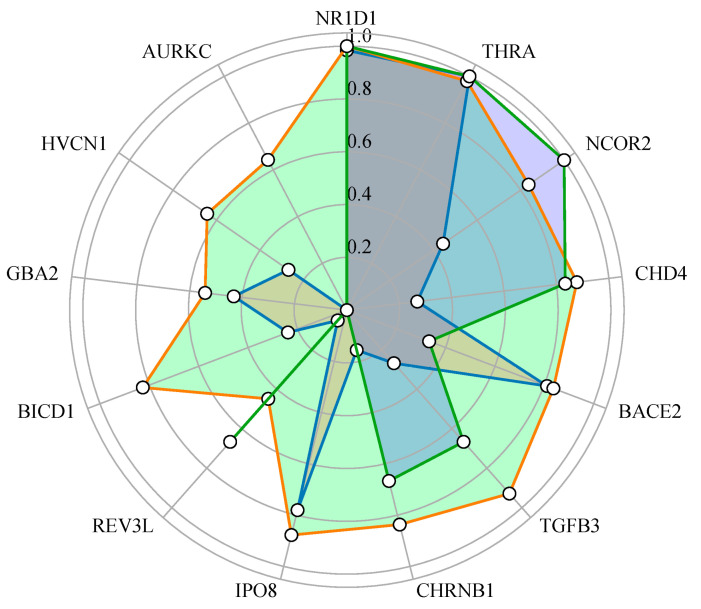
The radar plot of comprehensive scores for candidate genes. This plot shows the combined scores of three indicators for 13 candidate genes: coloc (orange), drug association (green), and SMR results (blue). Each axis represents a gene, and the score range is from 0 to 1. The colors represent the score distribution of specific indicators, while the gray shaded areas highlight genes with significant score changes (such as NR1D1, THRA, NCOR2).

In terms of drug targets and translational applications, we identified 13 druggable genes with significant causal relationships. Among these, protective genes such as CHD4 and HVCN1 are involved in anti-inflammatory and ion channel regulation pathways, while risk genes, such as GBA2, are implicated in lipid metabolism and cell adhesion processes. Based on the comprehensive SMR, colocalization, and PPI analysis results, the top five in the comprehensive score ranking are NR1D1, THRA, NCOR2, CHD4, and BACE2 (see Figure 11). It is particularly worth noting that the four genes, NR1D1, THRA, NCOR2, and CHD4, not only showed through the gene–drug interaction analysis that they had a highly reliable association with first-line migraine treatment drugs [42,43] (PPI score > 0.7), but there is also a potential synergistic effect with known migraine therapeutic targets, such as the CGRP pathway and the 5-HT receptor.

Furthermore, drug repurposing analysis identifies existing marketed drugs (e.g., GSK4112, Dextrothyroxine Sodium, benzbromarone, vorinostat) as potential migraine treatments, offering theoretical support. The corresponding candidate drugs converge three core pathophysiological axes in migraine (see Supplementary Table S10): (1) vascular dysregulation: TGF-β superfamily modulation (e.g., TGFB3) to balance tone, suppress pro-inflammatory (TGF-β1/2), and enhance anti-inflammatory (TGF-β3) signaling, improving endothelial function. (2) Neuroimmune inflammation: NRF2/HO-1 or complement inhibition (e.g., C5a blockers) to curb microglial activation and IL-1β release, blocking CSD cascades. (3) Neuronal hyperexcitability: BACE2 (Aβ inhibition + Nav1.6 stabilization) and nAChR modulation (e.g., varenicline) reduce CSD susceptibility. Multi-target agents (varenicline, SR9011) synergize via peripheral/central regulation. Epigenetic (HDAC inhibitors) and circadian (REV-ERBα agonists) mechanisms innovate beyond traditional paradigms.

However, BACE2’s controversial role and pan-TGF-β inhibitor toxicity require further study. And the current candidate drugs are primarily prioritized based on gene–drug interaction scores. Additional functional experiments are required to verify their effects on core migraine-related pathways (e.g., CGRP signaling and ion channel regulation) and to evaluate their therapeutic potential.

### 3.3. Cross-Study Comparison and Verification

Given that differences in outcome databases may affect the results, we employed the most recent and large-scale GWAS datasets to enhance the reliability of our findings. Compared with previous studies [44], we not only observed the repetition of some results, but also identified new potential targets, including REV3L and BACE2. Both this study and Zhang et al. (2024) [44] identified TGFB3 as a significant gene in both blood and brain tissue, and associated with lipid metabolism and insulin-like growth factor pathways [45,46]. Xiong et al., 2024 [47], using proteomic MR, discovered associations between proteins such as FCAR and UBE2L6 and migraines, and reported protein interactions between HBQ1 and topiramate (a preventive drug), but did not identify consistent results across different tissues. Similarly, we found that BACE2, CHRNB1, and TGFB3 had protein interactions with topiramate, but not such interactions with HBQ1. Although NCOR2 showed heterogeneity in different MR validation methods in this study, previous population studies have confirmed its association with migraine or epileptia-related phenotypes [48]. Our study shares a similar methodology to that of Sun et al. (2024) [49]. However, our study was based on large-scale data and systematically screened drug genes and candidate drugs, whereas Sun et al.’s (2024) [49] focused on identifying a single target, GSTM4, by integrating eQTL, pQTL, and GWAS data. In contrast to our study, which employed SMR and HEIDI as primary analytical methods, Sun et al. (2024) [49] used them only for validation purposes following initial target identification.

This study has several methodological strengths. We integrated multi-source GWASs and eQTL data, combined with SMR, multi-method MR validation, and strict horizontal pleiotropy control (*p* < 5 × 10^−8^), effectively enhancing the reliability of causal inference. However, due to the reliance on publicly aggregated data, differences in sample size, racial representativeness, and inconsistent data quality control standards may affect the validity of IVs [50]. For instance, stricter LD pruning or sample overlap control may lead to a reduction in instrumental variables, thereby affecting the significance of the results [51]. In addition, the sample size of this study is relatively large. Since Cochran’s Q test may show significant results due to the “large sample power” [52,53], we do not take the test results as the sole criterion. This might lead us to include more significant results. Furthermore, although confounding factors were excluded through the PheWAS database, the biological functions of some genetic variations have not been fully elucidated, and the risk of horizontal pleiotropy may still exist. In the future, it is necessary to further verify the robustness of causal associations based on larger-scale multi-ethnic cohorts, combined with fine phenotypic stratification and functional experiments [54].

### 3.4. Limitations and Future Research Directions

Complementing these mechanistic considerations is the need to address the limited generalizability of our findings, namely the overreliance on European-ancestry cohorts. Approximately 85–90% of GWASs for common traits, including migraine, focus on European populations, with cross-ancestry (African, East Asian) analyses hindered by small sample sizes and data heterogeneity. While key associated genes (e.g., “TGFB3”, “CHRNB1”) localize to conserved or low-variant regions, future work must integrate non-European cohorts via international collaborations and leverage cross-ethnic functional annotations (gnomAD, TOPMed) to evaluate locus-specific effects, strengthening the global relevance of our conclusions.

Moreover, our current druggability filter, focused on genes with established experimental or structural evidence, may overlook hidden opportunities. Of the 60 candidate genes, 47 could exhibit “cryptic” druggability via uncharacterized pockets or emerging modalities. To address this, we plan to systematically re-evaluate these targets using AlphaFold2-based conformational sampling to uncover novel binding sites. Concurrently, we will explore PROTACs (proteolysis-targeting chimeras) and RNA-targeting strategies to expand the druggability landscape beyond traditional pocket-dependent targets, thereby broadening therapeutic possibilities.

While expanding druggability horizons is critical, translating these targets into actionable therapies demands rigorous mechanistic validation. Although multi-omics (GWAS-eQTL-PheWAS) links genes like “NR1D1/CHD4” to migraine-related pathways (circadian disruption, neuroinflammation), it will be necessary in the future to conduct in vitro verification (e.g., neuronal excitability assays) to directly verify their causal role in migraine pathology mechanism. Moreover, further studies should be conducted to MR static nature inherently limits our ability to capture the “dynamic” gene expression shifts across migraine’s distinct phases. Prospective, time-stratified sampling of attacks remains logistically arduous and ethically complex, and publicly available resources like UK Biobank or GTEx lack annotations for migraine phases. To bridge this gap, future studies should integrate time-resolved transcriptomic profiling from migraine animal models paired with targeted clinical sampling during premonitory/ictal phases to dissect these patterns.

Another layer of complexity arises from the pleiotropic nature of many candidate genes and their associated drugs (e.g., “NR1D1”, “CHD4”, “BACE2”), which risk off-target effects. To mitigate this, we propose a multi-level optimization framework: (1) tissue-specific targeting (e.g., CGRP/TRPV1 nanoparticles or cell-type promoter-driven AAVs) to confine action to migraine-relevant tissues (trigeminal nerves, hypothalamus); (2) combinatorial therapy (synergistic drug pairing or dose fractionation) to enhance efficacy while reducing systemic toxicity; and (3) precision dosing aligned with circadian biology (e.g., REV-ERBα agonists) or real-time biomarkers (e.g., CGRP monitoring) to match migraine’s variability.

Taken together, these limitations underscore a clear roadmap for future research. Based on our findings, the following directions are recommended for future research:

(1) Conduct cross-ancestry validation studies to explore the impact of gene-environment interaction on migraine;
(2) Verify the functions of key genes (such as MICU1, UFL1, LY6G5C, PPP1CC) in the pathology of migraine through CRISPR/Cas9 or animal models;
(3) Develop targeted regulatory strategies for genes such as NR1D1, THRA, NCOR2, CHD4, and evaluate their roles in neuroinflammation and synaptic plasticity;
(4) Design multi-target combination therapies tailored to the different phases of migraine (e.g., acute vs. preventive), and integrate real-world adverse reaction data from monotherapies [55] to optimize treatment safety and efficacy.

In summary, this study provides a comprehensive analysis of the genetic-epigenetic molecular network of migraine. We identify multiple potential drug targets and repurposable drugs, offering new insights into the mechanisms of migraine and potential avenues for translational therapy [40]. Future research combining experimental verification and multi-omics analysis will be crucial to further elucidate migraine pathogenesis and promote precise diagnosis and treatment strategies.

## 4. Materials and Methods

### 4.1. Study Design

**Figure 12:**
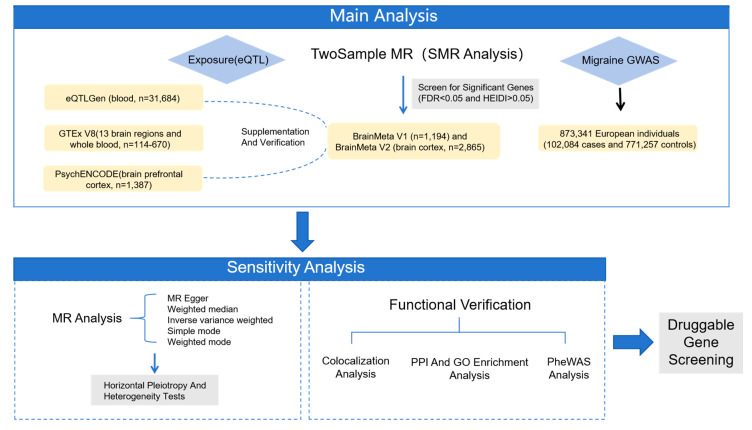
Design flowchart. Starting from gene expression data and disease GWAS data, through Mendelian randomization analysis, sensitivity analysis, and functional validation, the research process of ultimately screening out drug-target genes was performed.

This study is based on multiple publicly available databases, which are summarized in Supplementary Table S10. As shown in Figure 12, this study included relatively comprehensive cis-eQTL genetic variants as the IVs for gene expression. We performed SMR analysis for 13 regions in the brain and whole blood. We used the GTEx V8 (*n* = 114–670), PsychENCODE (*n* = 1387) project and eQTLGen (*n* = 31,684) summarized data as the main analysis. Due to the differences of various brain tissues, we also conducted subgroup analysis using BrainMeta v1 eQTL (*n* = 1194) and BrainMeta v2 (*n* = 2865) to supplement and verify the results. Multiple MR analysis was conducted on the key genes obtained from main analysis. To further examine the pleiotropy, linkage disequilibrium (LD), and heterogeneity at the target gene level, we utilized colocalization analysis, PPI, and GO enrichment analysis to evaluate the associations and mechanisms of action among genes. Finally, the clinical applicability of the candidate drug targets was evaluated through PheWAS.

### 4.2. Screening of Genetic Tools for Target Gene Expression

To identify common SNPs (MAF ≥ 1%) associated with the expression of migraine drug target genes in whole blood and the brain, we extracted publicly available eQTL data. The genetic tools for brain target gene expression originated from cis-eQTL summary data in the prefrontal cortex of the **PsychENCODE project** (*n* = 1387) and from whole blood and 13 brain regions of the **GTEx V8 project** (*n* = 114–670). The genetic tool for whole blood target gene expression was derived from the **eQTLGen Consortium** (https://www.eqtlgen.org/), which integrated 37 datasets covering 31,684 blood and peripheral blood mononuclear cell samples and reported 16,989 genes expressed in whole blood. All SNPs used in the above eQTL analysis originated from cis-regulatory regions (within 1 Mb of each gene), with a default *p*-value threshold of 5 × 10^−8^ (data sources are summarized in Supplementary Table S11).

### 4.3. Migraine GWAS Data

The aggregated statistical data of migraine outcomes were derived from the study by Hautakangas Heidi et al. [12]. This study integrated 873,341 individuals of European ancestry (102,084 cases and 771,257 controls) from five sets of European ancestry studies (see Supplementary Table S10).

### 4.4. Data Analysis

We employed the SMR method to evaluate the association between target gene expression levels (whole blood and brain eQTL) and migraine risk (GWAS). SMR is a Mendelian randomization tool based on aggregated data, which is used to explore causal relationships between genetic variations and phenotypes [16]. We used SMR Version 1.3.1 [16] with default settings, including a cis-eQTL *p*-value threshold of <5 × 10^−8^, MAF > 0.01, and excluded SNPs in strong LD (r^2^ > 0.9) with top eQTLs. Additionally, we removed SNPs with weak LD (r^2^ < 0.05) with top eQTLs. SMR results were derived from the most significant SNP per gene. To account for multiple testing, we used the Benjamini–Hochberg method [56] to correct for multiple comparisons and identify statistically significant associations. The HEIDI test was additionally conducted to assess horizontal pleiotropy. For genes that reached the corrected significance threshold (FDR < 0.05) and passed the HEIDI test (p_HEIDI > 0.05), the SMR site map was generated using the method described on the SMR webpage (https://yanglab.westlake.edu.cn/software/smr/#Overview, accessed on 13 April 2025). The effect direction on migraine was determined based on b_SMR, and the odds ratio (OR) and 95% confidence interval (CI) for the expression level of each significantly associated gene and its association with migraine risk were calculated, respectively.

### 4.5. Sensitivity Analysis of Key Target Genes

The robustness of causal relationships was tested by excluding reverse causality, horizontal pleiotropy, and uncertainty in colocalization. Reverse causality was excluded using the Steiger directionality test (*p* < 0.05). Horizontal pleiotropy was examined by testing the association of other genes within a 2 megabase (Mb) range of each genetic IV, and SMR analysis was used to determine if these adjacent genes were associated with migraine risk. Bayesian colocalization analysis was performed to assess whether two traits share a common causal variant, using the “coloc” package with default parameters (https://github.com/chr1swallace/coloc, accessed on 18 April 2025). This analysis calculates the posterior probabilities of five hypotheses (H0-H4) regarding whether a single causal variant is shared between the two traits. Hypothesis 4 (PPH4) proposes that both traits are associated with the genetic variant, and the associations are driven by the same causal variant. We used the coloc.abf algorithm to identify genes with PPH4 > 0.80 as determined by the algorithm.

The key genes obtained from SMR analysis were subjected to multiple MR analyses. Sensitivity analyses included horizontal pleiotropy, heterogeneity, and leave-one-out sensitivity tests. Horizontal pleiotropy was tested using MR–Egger regression [16,57]; significant intercept terms in MR–Egger analysis indicated horizontal pleiotropy [58]. Heterogeneity was quantified by calculating the Cochran’s Q statistic; a *p*-value < 0.05 for the Cochran’s Q test indicated significant heterogeneity [59]. Outliers detected by the MR pleiotropy residual sum and outlier test (MR-presso) were excluded, and the remaining IVs were reanalyzed [58]. The leave-one-out test examined the potential impact of a single SNP on causal effect estimation by sequentially excluding each SNP, calculating the meta-effect of the remaining SNPs, and assessing whether a single SNP drove the causal association [58].

### 4.6. External Validation Analysis

To conduct a more comprehensive summary analysis of eQTL data in brain tissue, SMR analysis was continued using the BrainMeta v1 eQTL summary data (*n* = 1194) by Qi et al. (2018) [60]. BrainMeta v1 eQTL is a group from MeC on GTEx Brain (GTEx Consortium 2017 Nature) [61], CMC [62], and ROSMAP (eQTL data from the meta-analysis of Nat Neurosci et al., 2017) [63]. Only SNPs within a 1Mb distance from each probe were available. In addition, chromosomal SMR subgroup analysis was conducted using BrainMeta v2 eQTL, and these data were from Qi et al. (2022) [64]. Cis-eQTL analysis of 16,704 eGenes in 2865 cerebral cortex samples from 2443 unrelated individuals of European origin Summary statistics (https://yanglab.westlake.edu.cn/software/smr/#eQTLsummarydata, accessed on 1 April 2025) was used to supplement the validation master analysis results.

### 4.7. Screening of Druggable Genes

The data of druggable genes mainly came from the study of Su et al. (2023) [32], integrating a total of 5883 druggable genes reviewed by DGIdb v4.2.0 (https://www.dgidb.org/) and Finan et al. (2017) [65]. In addition, 2532 overlapping druggable genes integrated by Kang-Fu Yin et al. (2024) [33] were also utilized for refinement and verification.

### 4.8. Candidate Gene Selection and Functional Enrichment Analysis

To conduct a more comprehensive screening of candidate genes, we used an online database resource, Pharos (https://pharos.nih.gov/), to evaluate the potential druggability of undruggable genes. To identify the role of key druggable genes in migraine, PPI was conducted via the STRING database. First-line intervention drugs for the attack and remission periods were obtained from clinical guidelines related to adult migraines [42,43], and the target genes associated with these different drug types were identified from the DrugBank database. Enrichment analysis was applied with significance thresholds of *p* ≤ 0.05 and Q ≤ 0.05 [66]. Candidate drug prediction was performed using DGIdb 5.0 (https://dgidb.org).

### 4.9. PheWAS Analysis of Candidate Gene

PheWAS analysis of candidate genes was conducted using the AstraZeneca PheWAS portal (https://www.azphewas.com), a publicly available repository of gene–phenotypic associations. These data were generated using sequencing data and phenotypic data collected in the UK. Based on the results of the phenotypic analysis, potential side effects or horizontal pleiotropy of the key targets were identified, which strengthened the validity of our analysis. The threshold for identifying genes with horizontal pleiotropy (i.e., associations with multiple phenotypes) in the PheWAS Manhattan plot was set at −log_10_(*p*-value) < 8.

## 5. Conclusions

This study provides a new direction for drug development for the precise treatment of migraine. It is recommended to give priority to the development of potential repurposable drugs based on the identified genes of NR1D1, THRA, NCOR2, and CHD4, such as GSK4112 (not approved), Dextrothyroxine Sodium, Benzbromarone, and Vorinostat. Particular attention should be paid to the roles of MICU1 (located on Chromosome 10), UFL1, LY6G5C (both located on Chromosome 6), and PPP1CC (located on Chromosome 12) in the pathological mechanism of migraine.

## Figures and Tables

**Table 1:** Overview of SMR main analysis results.

Source	Tissue	Gene
eQTLGen	Whole Blood	TJP2, B9D2, CCDC97, SNORA63, THRA, NR1D1, LY6G5C, RNFT2, PABPC4, ZC3H6, HRK, PPP1CC, SHISA5, UBE2D3-AS1, DNAJB12, RALGAPA1, CISD2, FAM8A1, PRR13, SFXN2, NCOR2, KLF10, GBA2, RGP1, PAM16, TGFB3, HVCN1, CHD4, ZMYND12, CCDC18-AS1, AURKC, RCCD1, RPS16 (novel transcript), RP11-252K23.2 (novel transcript), CTC-444N24.8 (novel transcript)
PsychENCODE	Prefrontal Cortex	KIAA0040, CFDP1, SLC9B1, CARF, ID4, CISD2, HMGXB3, RABAC1, RIMS1, UBE2D3-AS1, POLR1B, INPP5B, TREX1, TMA7, IPO8, SHISA5, EAPP, PACSIN3, SNX6, BICD1, RALGAPA1, CCDC18-AS1, ZMYND12
GTEx V8	Cerebellar Hemisphere	UFL1, LY6G5C, POC5, RCCD1, EXOSC5
	Cerebellum	UFL1, LY6G5C, RP11-753C18.8
	Whole Blood	UFL1, LY6G5C, FBXW8, LY6G5B, CTD-2509G16.2, RALGAPA1, PABPC4, HRK, ATP5SL

**Table 2:** SMR main analysis results of 13 druggable genes.

Tissue	Gene	Source	Chr	Top SNP	b_SMR	se_SMR	p_SMR	p_HEIDI	nsnp_HEIDI	FDR
Blood	AURKC	eQTLGen	19	rs2074858	−0.309	0.082	1.68 × 10^−4^	0.277	20	0.049
Blood	CHD4	eQTLGen	12	rs7969177	0.281	0.073	1.17 × 10^−4^	0.149	20	0.037
Blood	GBA2	eQTLGen	9	rs10814274	−0.068	0.017	7.54 × 10^−5^	0.173	20	0.030
Blood	HVCN1	eQTLGen	12	rs113511140	0.168	0.044	1.17 × 10^−4^	0.306	20	0.037
Blood	NCOR2	eQTLGen	12	rs1271309	−0.312	0.078	6.86 × 10^−5^	0.312	20	0.029
Blood	NR1D1	eQTLGen	17	rs883871	0.177	0.038	4.05 × 10^−6^	0.556	20	0.005
Blood	TGFB3	eQTLGen	14	rs146047341	−0.122	0.032	1.15 × 10^−4^	0.251	20	0.037
Blood	THRA	eQTLGen	17	rs883871	0.121	0.026	3.20 × 10^−6^	0.595	20	0.004
Brain—Prefrontal Cortex	BICD1	PsychENCODE	12	rs11051973	−0.087	0.023	1.13 × 10^−4^	0.213	20	0.038
Brain—Prefrontal Cortex	IPO8	PsychENCODE	12	rs61923728	−0.045	0.011	1.91 × 10^−5^	0.717	20	0.014
Brain—Hippocampus	BACE2	BrainMeta v1	21	rs914187	0.051	0.013	7.08 × 10^−5^	0.887	20	0.013
Brain—Cerebellum	CHRNB1	BrainMeta v1	17	rs60488855	−0.101	0.026	8.94 × 10^−5^	0.053	20	0.042
Brain—Cerebellum	REV3L	BrainMeta v2	6	rs12214097	0.052	0.015	3.53 × 10^−4^	0.683	20	0.046

Top SNP: the top associated cis-eQTL for the corresponding probe in the eQTL analysis; b_SMR: the causal effect size (Beta) calculated by SMR analysis; se_SMR: the Beta standard error calculated by SMR analysis; p_SMR: the *p*-value for SMR analysis; p_HEIDI: *p*-value from the HEIDI test (used to detect horizontal pleiotropy); nsnp_HEIDI: the number of SNPs used in HEIDI’s test; FDR: the Benjamini–Hochberg method was used to control the false discovery rate; FDR < 0.05 indicates statistical significance. Chr, chromosome; SNP, single-nucleotide polymorphism; SMR, summary data-based Mendelian randomization; QTL, quantitative trait loci; FDR, false discovery rate.

## Data Availability

The original contributions proposed in this research are included in this article. For further inquiries, please contact the corresponding authors directly.

## Supplementary Materials

The following supporting information can be downloaded at: https://www.mdpi.com/article/10.3390/molecules30193921/s1, Table S1. The main analysis results of eQTLGen; Table S2. The main analysis results of PsychENCODE; Table S3. The main analysis results of GTEx V8; Table S4 (a) Supplementary verification analysis of BrainMeta v1 and BrainMeta v2 (undruggable genes); Table S4 (b) Supplementary verification analysis of BrainMeta v1 and BrainMeta v2 (druggable genes); Table S5 (a) MR Multi-method validation of the significant results of the main analysis; Table S5 (b) MR Multi-method validation of druggable genes results; Table S6. Review of migraine novel development targets; Table S7. Exploration of the druggability of significant genes related to migraine targets; Table S8. The targets of first-line medication for migraine; Table S9. Summary table of candidate drugs; Table S10. Summary of mechanisms associated with migraine in candidate drugs; Table S11. Summary table of data sources.

## Abbreviations

The following abbreviations are used in this manuscript:

GWASs	Genome-wide association studies
eQTL	Expression quantitative trait loci
MR	Mendelian randomization
IV (IVs)	Instrumental variable (s)
SMR	Summary-based Mendelian randomization
HEIDI	Heterogeneity in dependent instruments
SNP(s)	Single nucleotide polymorphism(s)
PPI	Protein–protein interaction
PheWASs	Phenome-wide association studies
GO enrichment	Gene Ontology enrichment
LD	Linkage disequilibrium
